# Screening Primary-Care Patients Forgoing Health Care for Economic Reasons

**DOI:** 10.1371/journal.pone.0094006

**Published:** 2014-04-03

**Authors:** Patrick Bodenmann, Bernard Favrat, Hans Wolff, Idris Guessous, Francesco Panese, Lilli Herzig, Thomas Bischoff, Alejandra Casillas, Thomas Golano, Paul Vaucher

**Affiliations:** 1 Vulnerable Population Unit, Department of Ambulatory Care and Community Medicine, University of Lausanne, Lausanne, Switzerland; 2 Department of Ambulatory Care and Community Medicine, University of Lausanne, Lausanne, Switzerland; 3 Department of Community Medicine, Primary Care, and Emergency Medicine, University Hospital Geneva and Faculty of Medicine, University of Geneva, Geneva, Switzerland; 4 Institute of Social and Preventive Medicine (IUMSP), University Hospital Center and Faculty of Biology and Medicine, University of Lausanne, Lausanne, Switzerland; 5 Institute of the History of Medicine, Department of Ambulatory Care and Community Medicine, University of Lausanne, Lausanne, Switzerland; 6 Institute of General Medicine, Department of Ambulatory Care and Community Medicine, University of Lausanne, Lausanne, Switzerland; 7 Institute of Legal Medicine, School of Medicine, University of Geneva, Geneva, Switzerland; Cardiff University, United Kingdom

## Abstract

**Background:**

Growing social inequities have made it important for general practitioners to verify if patients can afford treatment and procedures. Incorporating social conditions into clinical decision-making allows general practitioners to address mismatches between patients' health-care needs and financial resources.

**Objectives:**

Identify a screening question to, indirectly, rule out patients' social risk of forgoing health care for economic reasons, and estimate prevalence of forgoing health care and the influence of physicians' attitudes toward deprivation.

**Design:**

Multicenter cross-sectional survey.

**Participants:**

Forty-seven general practitioners working in the French–speaking part of Switzerland enrolled a random sample of patients attending their private practices.

**Main Measures:**

Patients who had forgone health care were defined as those reporting a household member (including themselves) having forgone treatment for economic reasons during the previous 12 months, through a self-administered questionnaire. Patients were also asked about education and income levels, self-perceived social position, and deprivation levels.

**Key Results:**

Overall, 2,026 patients were included in the analysis; 10.7% (CI95% 9.4–12.1) reported a member of their household to have forgone health care during the 12 previous months. The question “Did you have difficulties paying your household bills during the last 12 months” performed better in identifying patients at risk of forgoing health care than a combination of four objective measures of socio-economic status (gender, age, education level, and income) (R^2^ = 0.184 vs. 0.083). This question effectively ruled out that patients had forgone health care, with a negative predictive value of 96%. Furthermore, for physicians who felt powerless in the face of deprivation, we observed an increase in the odds of patients forgoing health care of 1.5 times.

**Conclusion:**

General practitioners should systematically evaluate the socio-economic status of their patients. Asking patients whether they experience any difficulties in paying their bills is an effective means of identifying patients who might forgo health care.

## Introduction

Wealth and health disparities exist worldwide and the gap is widening even in the most developed countries.[1]–[4] These disparities are being addressed as a major public-health concern.[5], [6] Policy makers are tackling the problem from an upstream reform perspective (e.g. improvement of education levels, income redistribution, and universal health-insurance coverage). Nevertheless, addressing existing disparities and their immediate consequences remains a crucial task and requires the active participation of health-care providers (downstream perspective).[7]

General practitioners (GPs) can address mismatches between patients' health-care needs and their financial resources[8] by incorporating an awareness of social vulnerability[7] into their clinical decision making. This however requires physicians to be able to overcome barriers to discussing financial issues, particularly by firstly ruling out the risk of forgoing health care for economic reasons, a risk which is important even in Switzerland,[9]–[11] a country with a universal and compulsory private health insurance coverage system that includes subsidies for citizens on low-incomes. This risk of forgoing care also impacts the decision to undergo clinical encounters and adherence to treatment,[12] and may result in unfavorable health outcomes.[13] Many GPs possibly fail to screen for this risk as they already face multiple and complex, competing demands on their time during visits; they might also feel uncomfortable discussing financial matters with their patients — many providers do not feel trained to discuss such problems, especially when they do not believe they can offer any satisfactory solution.[14] Furthermore, current questionnaires addressing these issues are seldom useful as the questions are frequently country-specific[15] and/or time consuming for routine use.[16] Thus, a rapid screening tool to identify a patient's risk of forgoing health care, without imposing on his/her sense of security or comfort during the patient-provider encounter, would prove useful for effective patient care in everyday medical practice.

This study aims to identify the optimal single field screening question for ruling out patients' risk of forgoing health care for economic reasons. We also wanted to estimate the prevalence of forgoing health care in primary care, and test the influence of physicians' attitudes toward deprivation.

## Methods

### Recruitment and Data Collection

This survey was nested in a study designed to investigate deprivation in patients visiting primary-care physicians.[16] Two thousand and twenty-five randomly selected patients were recruited from a convenience sample of 47 GPs working in urban, rural, and suburban private practices, in the western, French-speaking region of Switzerland. This population corresponds to seven of the 26 Swiss states, comprised of 1.6 million French speakers (20% of the total national population). Data were collected from September 2010 to February 2011. Randomization procedure was used to identify one of 10–12 visits per half-day (depending on the information provided by each GP). GPs were then provided with individualized calendars that indicated which patients to recruit. Inclusion criteria were: having a primary care visit at the selected practice during the day, being over 16 years of age, and being able to understand either French, German, Italian (the three national languages), or English. The self-administered questionnaire was independently completed in the waiting room, separate from the GP, and sent to a research psychologist within seven days. Any missing data were completed by the research psychologist during a follow-up telephone call. GPs were blinded to their patients' responses. Each physician stopped recruitment once he or she had included 50 random patients, or after 12 weeks.

### Primary Measure — Screening for Forgoing Health Care for Economic Reasons

Each household member's exposure to social risk-factors affects all other members,[17] which may influence access to care.[18], [19] We therefore chose to ask about restricted access to health care at the household level. Patients were asked: “During the last 12 months, has a member of your household not sought treatment (dentist, doctor, or buying medication) because you didn't have enough money?”

### Other Measures — Patient Determinants

Other self-reported and self-perceived subjective measures included social determinants of state-of-deprivation,[16] social position,[20] and health status.[21] These were compared to more objective social determinants related to patients' socio-economic status (SES): nationality, age, gender, education level, household's source of income, household's overall income level, and number of household members.[22] To explore subjective social status related to deprivation we used questions from the DiPCare-Q. Details on psychometric properties for each question are provided in a separate paper.[16] Individuals' daily available financial resources were calculated by subtracting the subsistence level family income figure, defined by the modified equivalence scale,[23] from the gross, daily household income, and dividing it by the number of household members.

### Other Measures — Physician-level Determinants

All participating physicians were asked to complete a questionnaire regarding their perceived role in handling social disparities for the patient care they provide. They were questioned about the attention they paid to issues of deprivation, about stereotypes related to deprivation, about their feelings of gratification, frustration, overwork, or powerlessness when facing patient deprivation, whether deprivation influenced the time they spent with a patient, the type of medical investigation or the doctor–patient relationship, whether they thought patients wanted to talk about deprivation with them or not, and whether they thought investigating deprivation was part of their role as a GP.

### Statistical Methods

From previous observations,[9], [22] we estimated that 15% of patients had restricted their access to health care during the previous 12 months. With a significance level set at 0.05 and powered to 0.8, we designed the study to detect a two-fold odds ratio for an exposure that would be present in 10% of cases and 5% of controls. Estimated analytic sample size was 1,888 patients. Given that we predicted 5% of questionnaires would have a missing response regarding restricted household access to health care, the total number of patients estimated for inclusion was 2,000.

From known social determinants of health, we searched for those that were linked to the renunciation of health care. Student's t-test (or Wilcoxon's signed-rank test if distributions were not normal) was used for comparison of means (continuous variables), and a Chi^2^ test for comparison of percentages (categorical variables). We accounted for patients who did not answer a question by creating a “missing” category for all studied determinants. Crude and adjusted ORs were calculated for education level, income, nationality, number of household members, subjective social status, and each of the 16 questions included in the DiPCare-Q.[16] The clustering effect of physicians' attitudes was evaluated by measuring their influence on forgoing health care at the physician level using a random effect model. Two models were used to adjust ORs for confounders. The first adjusted for age, gender, health status, and the clustering effect at a physician level, using generalized estimate equations with robust standard error. The second used logistic regression adjusting for age, gender, health status, and physicians' characteristics. The single question to retain was the one with the highest coefficient of determination (R^2^). Significant level was set at p<0.05. Bonferroni adjustment for multiple testing was not used, as factors were highly correlated to one another and their independence could not be assumed. Linearity of categorical variables was tested comparing logistic regression models with values first entered as dichotomized values, then as integers. Linearity was assumed when the likelihood ratio test between models showed no significant difference (p≥0.05). All statistical analysis was carried out with STATA 12.0, Statacorp, College Station, Texas, USA.

### Ethics Statement

Patients were given oral and written information concerning the study, prior to the time they spent with their physician, by the medical secretary. They were clearly told that participation was voluntary and that refusing to participate would have no consequence for the care provided by their physician who remained blinded to their participation. They were clearly informed that handing back the questionnaire in a sealed envelope after their visit meant that they agreed to participate. The study was conducted in accordance with the principles expressed in the Declaration of Helsinki. The study was approved by the Ethical Committee of the Canton of Vaud under reference number 157/10. Data from this study is publicly available on Dryad (doi:10.5061/dryad.2mg29).

## Results

**Figure 1:**
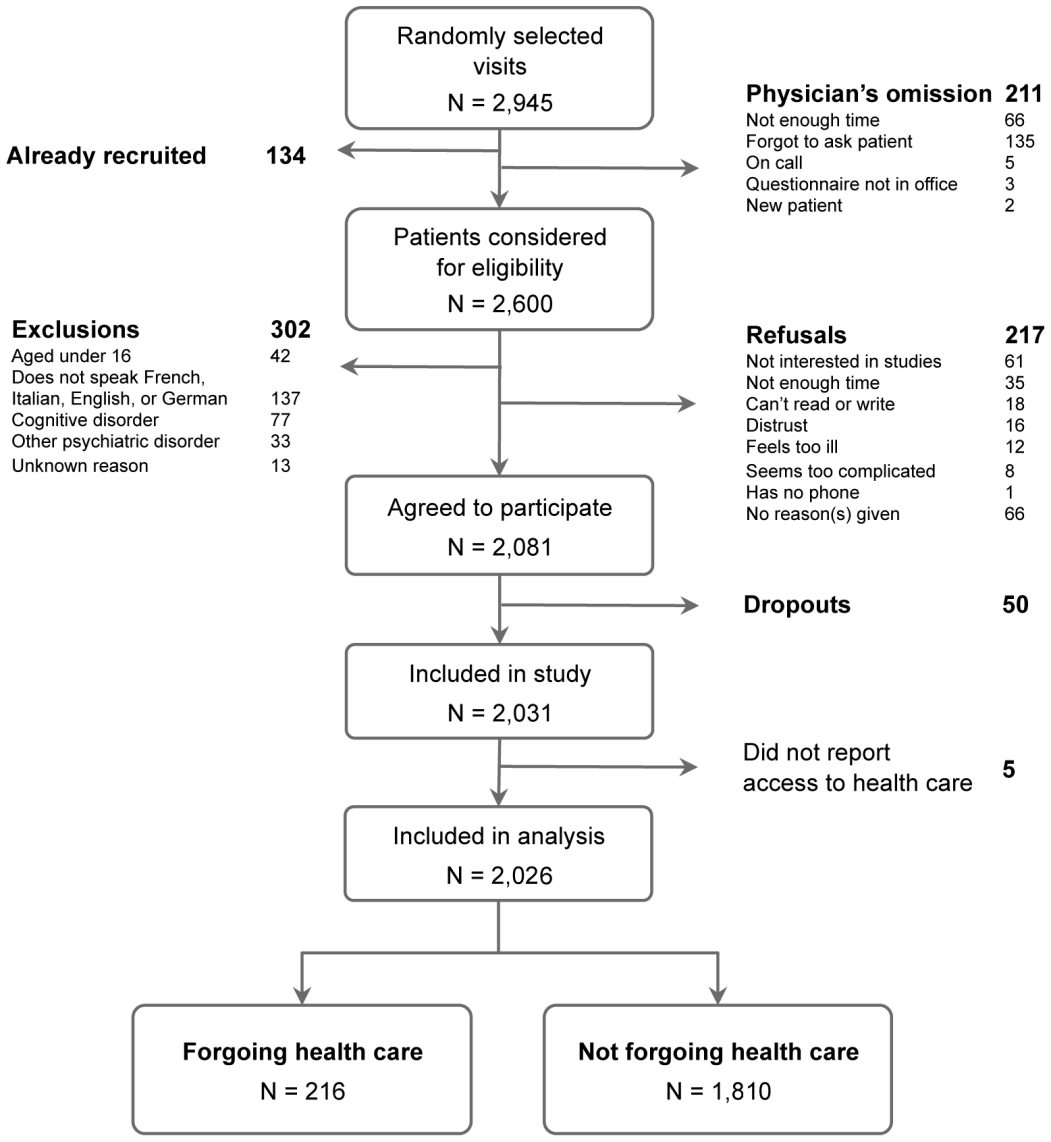
Flow chart of the study population selection including patient recruitment, exclusion criteria, and refusals.

From the 2,811 randomly selected patients (2,945 visits), data from 2,026 patients were included in our analysis (inclusion rate of 72.1%). Reasons for exclusion are provided in Figure 1. Of the physicians from whom we recruited patients, 72.3% were male, mean age was 54 (SD 9 years), and average duration of practice was 18.9 years (SD 10.6 years). There were relatively similar proportions of urban- (31.8%), rural- (31.8%), and suburban (36.4%) area practices. In many questionnaires, some isolated questions remained unanswered. The most frequent unanswered question concerned patients' “Level of income” (n = 343, 16.9%) followed by patients' level of education (n = 88, 4.3%). The proportion of patients who failed to provide an answer was nevertheless similar between those who reported forgoing health and those who did not for all determinants but one (subjective social status reported by patients; Tables 1 & 2).

**Table 1:** Socio-economic characteristics of studied population and univariate association to forgoing health care.

	All patients	Access to health care		
	n = 2,026	Did forgo health care n = 216	Did not forgo health care n = 1,810	p-value
Age (years)				p<0.001
* 16–24*	6.0% (122)	7.9% (17)	5.8% (105)	
* 25–39*	14.9% (301)	22.2% (48)	14.0% (253)	
* 40–64*	43.0% (872)	53.2% (115)	41.8% (757)	
* 65–79*	24.9% (505)	11.6% (25)	26.5% (480)	
* ≥80*	9.0% (182)	1.4% (3)	9.9% (179)	
* Missing*	2.2% (44)	3.7% (8)	2.0% (36)	*P = 0.102*
Gender				P = 0.427
* Male*	40.4% (818)	36.6% (79)	40.8% (739)	
* Female*	57.8% (1172)	61.1% (132)	57.5% (1040)	
* Missing*	1.8% (36)	2.3% (5)	1.7% (31)	*P = 0.527*
Education				P = 0.012
* Incomplete compulsory schooling*	4.9% (99)	7.9% (17)	4.5% (82)	
* Complete compulsory schooling*	21.4% (433)	24.1% (52)	21.0% (381)	
* General vocational training*	46.8% (949)	45.8% (99)	47.0% (850)	
* Higher education*	22.6% (457)	15.7% (34)	23.4% (423)	
* Missing*	4.3% (88)	6.5% (14)	4.1% (74)	*P = 0.103*
Nationality‡				
* Swiss*	79.9% (1,619)	70.4% (152)	81.0% (1,467)	P<0.001
* European*	21.8% (441)	25.5% (55)	21.3% (386)	P = 0.164
* Other*	3.5% (70)	7.4% (16)	3.0% (54)	P = 0.001
* Missing*	1.8% (36)	2.3% (5)	1.7% (31)	*P = 0.527*
Reported source of household income‡				
* Wage*	50.6% (1,026)	63.0% (136)	49.2% (890)	P<0.001
* Self-employed salary*	7.1% (144)	6.5% (14)	7.2% (130)	P = 0.705
* Retirement pension*	35.6% (721)	15.3% (33)	38.0% (688)	P<0.001
* Invalidity insurance pension*	9.1% (184)	12.5% (27)	8.7% (157)	P = 0.064
* Unemployment benefit*	3.2% (65)	11.1% (24)	2.3% (41)	P<0.001
* Social welfare*	4.2% (85)	8.8% (19)	3.6% (66)	P<0.001
* Loss-of-income insurance*	2.5% (50)	3.2% (7)	2.4% (43)	P = 0.439
* Widow's pension*	3.6% (74)	0.9% (2)	4.0% (72)	P = 0.024
* Alimony (divorce)*	2.5% (51)	1.4% (3)	2.6% (48)	P = 0.263
* Study grant*	0.8% (17)	2.3% (5)	0.7% (12)	P = 0.012
* Assets (property, shares)*	8.0% (163)	2.3% (5)	8.7% (158)	P = 0.001
* Parents/family/friends*	4.4% (90)	5.6% (12)	4.3% (78)	P = 0.401
* Missing*	2.5% (50)	2.3% (5)	2.5% (45)	*P = 0.878*
Income				
* Individual's daily available financial resources ^†^*				P<0.001
* < 0 CHF*	6.2% (126)	12.0% (26)	5.5% (100)	
* 0–19 CHF*	16.1% (326)	24.5% (53)	15.1% (273)	
* 20–49 CHF*	21.7% (439)	26.4% (57)	21.1% (382)	
* 50–99 CHF*	22.5% (455)	19.0% (41)	22.9% (414)	
* ≥100 CHF*	16.6% (337)	5.6% (12)	18.0% (325)	
* Missing*	16.9% (343)	12.5% (27)	17.5% (316)	*P = 0.066*
* Relative poverty* *				P<0.001
* Above*	76.8% (1557)	75.5% (163)	77.0% (1394)	
* Under*	6.2% (126)	12.0% (26)	5.5% (100)	
* Missing*	16.9% (343)	12.5% (27)	17.5% (316)	*P = 0.066*

*Not included in model 1 as this variable was highly correlated to a similar factor. *†* in Swiss Francs (CHF 1  =  US$ 1.10).

‡ More than one response was possible.

**Table 2:** Subjective social determinants and association to forgoing health care.

	All patients	Access to health care		
	N = 2,026	Did forgo health care n = 216	Did not forgo health care n = 1,810	p-value
Material deprivation factors				
* Difficulties paying bills*	25.8% (523)	74.1% (160)	20.1% (363)	P<0.001
* Need to borrow money for daily expenses*	13.8% (279)	44.0% (95)	10.2% (184)	P<0.001
* Scared of losing housing*	4.7% (96)	14.8% (32)	3.5% (64)	P<0.001
* Can't afford clothes*	17.4% (353)	54.6% (118)	13.0% (235)	P<0.001
* Can't afford furniture*	19.3% (392)	56.9% (123)	14.9% (269)	P<0.001
* Not enough to eat at home*	5.7% (115)	18.5% (40)	4.1% (75)	P<0.001
* Difficulties reimbursing loan(s)*	14.1% (286)	47.2% (102)	10.2% (184)	P<0.001
Social deprivation factors				
* No holidays*	39.5% (800)	63.9% (138)	36.6% (662)	P<0.001
* No evening(s) spent with family or friends*	16.3% (331/2,020)	35.2% (76)	14.1% (255)	P<0.001
* No cultural activities*	50.0% (1,013)	70.8% (153)	47.5% (860)	P<0.001
* No access to the Internet*	25.8% (523)	22.7% (49)	26.2% (474)	P = 0.263
* No one to turn to for material support*	32.1% (644)	46.5% (100)	30.4% (544)	P<0.001
Health deprivation factors				
* Physical handicap*	21.4% (433)	28.2% (61)	20.5% (372)	P = 0.005
* Psychic handicap*	16.9% (342)	31.9% (69)	15.1% (273)	P<0.001
* Addiction*	5.3% (108)	8.8% (19)	4.9% (89)	P = 0.016
Subjective social status				
* Patient's evaluation*				P<0.001
* 8–10 (highest)*	19.1% (387)	4.2% (9)	20.9% (378)	
* 6–7*	34.9% (706)	16.7% (36)	37.0% (670)	
* 4–5*	35.0% (710)	47.7% (103)	33.5% (607)	
* 1–3 (lowest)*	8.5% (173)	26.8% (58)	6.4% (115)	
* Missing*	2.5% (50)	4.6% (10)	2.2% (40)	*P = 0.030*
* Physician's evaluation*				P<0.001
* 8–10 (highest)*	32.6% (661)	19.4% (42)	34.2% (619)	
* 6–7*	33.2% (672)	31.0% (67)	33.4% (605)	
* 4–5*	22.7% (459)	29.6% (64)	21.8% (395)	
* 1–3 (lowest)*	10.4% (210)	18.1% (39)	9.5% (171)	
* Missing*	1.2% (24)	1.9% (4)	1.1% (20)	*P = 0.338*
Health				
* EQ5D_Europe_*				P<0.001
* 100 (perfect health)*	20.1% (407)	8.8% (19)	21.4% (388)	
* 75–99*	31.3% (635)	24.1% (52)	32.2% (583)	
* 50–74*	37.0% (749)	48.1% (104)	35.6% (645)	
* <50*	9.4% (191)	16.2% (35)	8.6% (156)	
* Missing*	2.2% (44)	2.8% (6)	2.1% (28)	*P = 0.183*
* VAS value EQ5D*				P<0.001
* 76–100*	41.3% (836)	29.6% (64)	42.7% (772)	
* 51–75*	31.4% (637)	31.9% (69)	31.4% (568)	
* 26–50*	21.5% (436)	28.7% (62)	20.7% (374)	
* 0–25*	2.4% (49)	4.6% (10)	2.1% (39)	
* Missing*	3.4% (68)	5.1% (11)	3.1% (57)	*P = 0.134*

For patients consulting their GP in western Switzerland, period prevalence of forgoing health care during the previous 12 months was 10.7% (95%CI, 9.4–12.1). Compared to other patients, those whose household members had forgone health care due to out-of-pocket expenses had a lower household-income, were younger, were more likely to suffer from poverty, were more likely to receive income from social- or unemployment welfare, a study grant, or a wage, but were less likely to be from a household with sources of income from retirement, private assets, or a widow's pension, or to have Swiss nationality (Table 1). Forgoing health care was associated with each of the 16 items used in the deprivation index DIPCare-Q,[16] the material index, subjective social status evaluated by patients or physicians, and health status (Table 2). However, not having access to the Internet was only associated with forgoing health care for patients older than 65. Physicians' self-perceived role was also associated with patient risk of forgoing health care. Adjusting for other factors, forgoing health care was less likely for patients who were seen by physicians who perceived that their role was to care for deprived patients (OR_adj_ = 0.68; CI95% 0.47 to 0.97), or by physicians who stated that they forgo additional investigation or expensive treatments when appropriate (OR_adj_ = 0.52; CI95% 0.33 to 0.81). On the other hand, physicians who stated that they feel powerless when facing patient deprivation were more likely to have patients forgo health care (OR_adj_ = 1.5; CI95% 1.1 to 2.1). These three factors were accounted for when measuring the magnitude of each question about forgoing health care (Table 3, Model 2).

**Table 3:** Odds of forgoing health care (n = 2,026).

	R^2^ % explained variance	Odds ratio		
		Unadjusted	Adjusted Model 1*	Adjusted Model 2†
**Objective determinants**				
Gender (male)‡	0.1%	0.84	0.91 [0.65 to 1.3]	0.91 [0.67 to 1.2]
Age (*≥*65 years)‡	4.0%	0.26	0.26 [0.17 to 0.41]	0.25 [0.17 to 0.38]
Being non-Swiss‡	0.9%	1.8	1.5 [1.0 to 2.2]	1.5 [1.1 to 2.1]
Education level	0.9%			
* Higher education*		1 (Ref.)	1 (Ref.)	1 (Ref.)
* General vocational training*		1.4	1.4 [1.0 to 2.0]	1.5 [0.95 to 2.2]
* Complete compulsory schooling*		1.7	1.8 [1.1 to 2.9]	1.9 [1.2 to 3.0]
* Incomplete compulsory schooling*		2.6	2.2 [1.2 to 4.0]	2.1 [1.1 to 4.2]
* Missing*		2.4	3.0 [1.4 to 6.5]	3.2 [1.3 to 7.5]
Available daily income (CHF) **	3.6%			
* ≥100.-*		1 (Ref.)	1 (Ref.)	1 (Ref.)
* 55–99*		2.7	2.7 [1.5 to 4.9]	2.8 [1.4 to 5.5]
* 20–49*		4.0	3.7 [2.0 to 6.6]	3.8 [2.0 to 7.3|
* 0–19*		5.3	5.4 [2.9 to 10.0]	5.7 [2.9 to 11.0]
* <0*		7.0	6.2 [2.8 to 13.4]	6.3 [3.0 to 13.2]
* Missing*		2.3	2.3 [1.3 to 4.1]	0.0 [0.0 to 0.0]
**Subjective determinants**				
Material deprivation factors				
* Difficulties paying bills*	18.4%	11.4	8.9 [6.5 to 12.0]	8.8 [6.3 to 12.4]
* Need to borrow money for daily expenses*	10.0%	6.9	5.2 [4.0 to 6.7]	5.2 [3.7 to 7.2]
* Scared of losing housing*	2.8%	4.7	3.2 [2.1 to 4.7]	3.0 [1.9 to 4.9]
* Can't afford clothes*	13.0%	8.1	6.2 [4.5 to 8.5|	6.3 [4.6 to 8.7]
* Can't afford furniture*	12.6%	7.6	5.7 [4.2 to 7.6]	5.7 [4.1 to 7.8]
* Not enough to eat at home*	3.8%	5.3	3.8 [2.6 to 5.6]	3.7 [2.4 to 5.7]
* Difficulties reimbursing loan(s)*	11.7%	7.9	5.7 [4.4 to 7.5]	5.6 [4.1 to 7.8]
Social deprivation factors				
* No holidays*	4.3%	3.1	3.2 [2.4 to 4.3]	3.3 [2.4 to 4.5]
* No evening(s) spent with family or friends*	3.8%	3.3	3.2 [2.4 to 4.2]	3.2 [2.3 to 4.5]
* No cultural activities*	3.1%	2.7	2.7 [2.0 to 3.5]	2.7 [1.9 to 3.7]
* No access to the Internet*				
* <65 years of age*	0.1%	1.2	0.94 [0.60 to 1.5]	0.94 [0.58 to 1.5]
* ≥65 years of age*	1.4%	2.0	2.6 [1.1 to 6.2]	2.7 [1.3 to 5.9]
* No one to turn to for material support*	1.6%	2.0	2.1 [1.6 to 2.7]	2.1 [1.5 to 2.8]
Subjective social status	10.7%			
* 8–10 (highest)*		1 (Ref.)	1 (Ref.)	1 (Ref.)
* 6–7*		2.3	1.7 [0.85 to 3.6]	1.8 [0.85 to 3.8]
* 4–5*		7.1	5.3 [2.7 to 10.3]	5.3 [2.6 to 10.7]
* 1–3 (lowest)*		21.2	14.6 [7.5 to 28.4]	14.7 [6.9 to 31.2]
* Missing*		10.5	10.4 [3.4 to 31.6]	10.9 [3.5 to 34.4]
**Health status**				
EQ5D_EU_	3.0%			
* 100 (perfect health)*		1 (Ref.)	1 (Ref.)	1 (Ref.)
* 75–99*		1.8	1.8 [1.1 to 3.1]	1.8 [1.1 to 3.2]
* 50–74*		3.3	3.4 [2.3 to 5.2]	3.4 [2.0 to 5.6]
* <50*		4.6	4.3 [2.8 to 6.6]	4.3 [2.4 to 7.9]
* Missing*		3.2	2.9 [0.91 to 9.2]	3.2 [1.1 to 9.2]

*In model 1, determinants were adjusted for age, gender, health status, and the clustering effect at a physician level.

† In model 2, determinants were adjusted for age, gender, health status, physician does not endorse social role, physician seldom forgoes expensive treatment or investigations for deprived patients, and physician feels powerless when facing deprivation.

‡ Missing data was not associated to forgoing health care.

**CHF 1  =  US$ 1.1.

**Figure 2:**
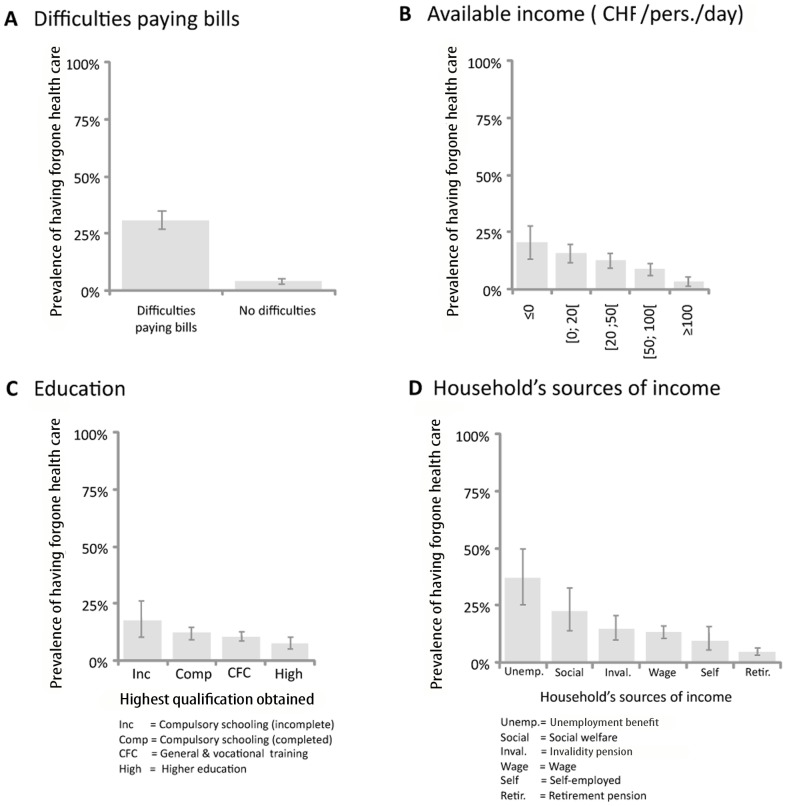
Prevalence of patients affected by forgoing healthcare within subpopulations. Determinants are (A) their household's ability to pay bills, (B) their daily available income, (C) their level of education, or (D) their household's sources of income. Intervals correspond to CI95%. CHF 1  =  US$ 1.10.

The question which was best associated with the risk of forgoing health care (Table 3) was the first question from the DiPCare-Q[16]: “During the last 12 months, have you had trouble paying your household bills (taxes, insurance, telephone, electricity, credit cards, etc.)?” (Figure 2). Compared to those who responded negatively (n = 1,503), those who replied positively (n = 523) were 11.4 times more likely (95%CI 8.2 to 15.8) to have forgone health care.

This single subjective question alone was a better determinant of forgoing health care than a combination of four common objective determinants: gender, age, education level, and level of income (R^2^ = 0.184 vs. 0.083). Finally, this question has a sensitivity of 74.1%, a specificity of 79.9%, and a negative predictive value of 96.3% in detecting patients who report having forgone health care.

## Discussion

In our study, around 1 in 10 patients (10.7%) were affected by out-of-pocket health-care expenses and had seen one of their household members forgo health care during the 12 previous months. GPs could help diminish the health burden caused by existing disparities if only they could identify these patients more easily.[24] This study reveals a simple way to help GPs screen for and rule out the risk of forgoing health care for economic reasons: ask patients whether their household has had difficulties paying its bills. Asking directly if a patient has forgone health care for economic reasons may lead to an important underestimation because of social desirability bias and stigma. The patient might fear that his GP will not care for him if he cannot afford to pay.[25], [26] Furthermore our study shows that this single question performed better than a combination of information from objective socio-economic-status markers. Interestingly, physicians' attitudes and beliefs concerning their role in caring for deprived patients may also have an impact on whether patients forgo health care.

Even in a universal and compulsory private health insurance coverage system with subsidies for individuals on low incomes, the prevalence of patients forgoing health care was high, similar to the results of a national telephone survey[10] but slightly lower than the prevalence reported in Geneva's urban-population-based surveys.[9], [11] International comparisons are difficult because of the multitude of factors related to national health systems, but a recent American study did show that 10% of US families did not obtain the care they needed due to the financial burden such care entailed.[19] Thus, one can see that cost-sharing health policies generate health disparities in similar proportions in other countries.[22]

Identifying patients facing financial difficulties and economic hardship is an important challenge for GPs who generally do not assess patients for problems related to out-of-pocket health-care costs[24], [27], [28]: previously noted obstacles to such an assessment are not feeling at ease discussing financial issues, insufficient time, and a lack of solutions for a problem perceived as unsolvable. Yet while, in a study by Alexander et al,[24] patients (305/484, 63%) and physicians (105/133, 79%) believed that discussion of out-of-pocket costs was important, these discussions only occurred infrequently (35% for physicians and 15% for patients). In our study, it was seen that using a simple screening question can reasonably rule out the risk of forgoing health care for economic reasons. This single question is easy to use because it is less stigmatizing than asking about actual income. It is also not country-specific and performs better than a combination of information from objective and individual social-economic predictors (NPV 96%). That said, asking patients directly about the financial consequences of health-care expenses might be more relevant. This is especially the case when planning expensive investigations or treatments. Asking about difficulties paying bills is probably more relevant if we are interested in knowing if a patient is at risk of forgoing health care due to difficulties that they have not yet been confronted with. If patients are positive for this single screening question, this screen would encourage patients and physicians to engage in a more in-depth discussion about out-of-pocket costs, individualized plans of treatment depending on patient circumstances, and the consequences of forgoing health care for economic reasons.

Studies have shown that non-adherence to medication due to cost pressures is positively influenced by a trustful physician–patient relationship,[29] demonstrating that medication underuse is not simply an economic issue. Studies have described the communication skills needed to discuss health-care costs with patients, skills which serve to improve shared decision making, negotiation, and the consideration of alternatives.[30] Our study highlights that physicians' attitudes toward discussing health-care costs can affect patient access to health care; perhaps patients can more easily open up regarding the burdens and realities of health-care costs when being treated by a more sensitive and empathetic physician.

To our knowledge, this is only the second study to focus on a single question to rule out patients' risk of forgoing health care for economic reasons in the GP-practice setting, the first being a pilot study in Canada by Brcic et al.[31] That pilot study also found that a question regarding making ends meet was the best indicator of poverty. The present paper is the first in which the prevalence of forgoing health care has been evaluated in GPs' practices in Switzerland, a country with a universal and compulsory private health insurance coverage system. Furthermore, and as shown by Gruen[32] and Alexander, [24], [27] this study underlines the importance of physicians' attitudes toward patients' economic hardship and risk of forgoing health care.

Nevertheless, this single question should be prospectively validated in different health-care systems. One limitation is that we did not ask patients about the medical problems linked to the health care that had been forgone for economic reasons (to better define these as major or minor medical problems). This should be included in future studies. We attempted to minimize response bias related to patient discomfort by administering a self-report questionnaire in the waiting room, away from the presence of the physician. However, despite these best efforts the true prevalence of forgoing health care for economic reasons may be higher than the results presented here, due to factors such as social stigma. Finally, having patients self-report their income may be a fairly inaccurate means of determining true household income. Our approach was, however, pragmatic; physicians can only rely on the answers provided to them by their patients, even if these are sometimes inaccurate. It can, however, be argued that this pragmatic approach might wrongly assume that patients would have also failed to respond to the same questions had they been asked by their physician. The reasons for which patients chose not to respond to isolated questions remain unknown. Patients might have missed some unintentionally, or been unable to provide an answer, or had difficulties understanding the question clearly. They may even simply have refused to share certain information they believed to be private. Therefore, the external validity of the value of missing answers in detecting the risk of forgoing health care is limited and should be interpreted with care.

## Conclusion

The physician plays an important role in identifying and preventing high-risk patients (low socio-economic status, multi-morbid, elderly) from forgoing health care.

Asking all patients about their ability to pay their household bills, in order to rule out if patients are at risk, is a simple, generalizable, and effective way of screening for financially vulnerable patients. If positive, this question should trigger a conversation on a medical-care issue that is frequently neglected; thus offering the patient and physician an opportunity to share in an open conversation regarding an appropriate, realistic, and patient-tailored treatment plan.

## References

[1] Marmot M, Friel S, Bell R, Houweling TAJ, Taylor S (2008) Closing the gap in a generation: health equity through action on the social determinants of health. Lancet 372: 1661–1669. 18994664 10.1016/S0140-6736(08)61690-6

[2] Betancourt JR, Green AR, King RR, Tan-McGrory A, Cervantes M, et al. (2011) Improving Quality and Achieving Equity: A Guide for Hospital Leaders. Massachusetts General Hospital: The Disparities Solutions Center website, www2 massgeneral org/disparitiessolutions/guidehtml Accessed March 3.

[3] Carter-Pokras O, Baquet C (2002) What is a “health disparity”? Public health reports 117: 426–434. 12500958 10.1093/phr/117.5.426 PMC1497467

[4] Taylor S (2009) Wealth, health and equity: convergence to divergence in late 20th century globalization. British Medical Bulletin 91: 29–48. 19654137 10.1093/bmb/ldp024

[5] Östlin P, Schrecker T, Sadana R, Bonnefoy J, Gilson L, et al (2011) Priorities for Research on Equity and Health: Towards an Equity-Focused Health Research Agenda. PLoS Med 8: e1001115. 22069378 10.1371/journal.pmed.1001115 PMC3206017

[6] Krieger N (2001) Theories for social epidemiology in the 21st century: an ecosocial perspective. Int J Epidemiol 30: 668–677. 11511581 10.1093/ije/30.4.668

[7] Franks P, Fiscella K (2008) Reducing disparities downstream: prospects and challenges. J Gen Intern Med 23: 672–677. 18214626 10.1007/s11606-008-0509-0 PMC2324139

[8] Bloch G, Rozmovits L, Giambrone B (2011) Barriers to primary care responsiveness to poverty as a risk factor for health. BMC Fam Pract 12: 62. 21714925 10.1186/1471-2296-12-62 PMC3135547

[9] Wolff H, Gaspoz JM, Guessous I (2011) Health care renunciation for economic reasons in Switzerland. Swiss Med Wkly 141: w13165. 21337175 10.4414/smw.2011.13165

[10] Schoen C, Osborn R, Squires D, Doty MM, Pierson R, et al (2010) How health insurance design affects access to care and costs, by income, in eleven countries. Health Aff (Millwood) 29: 2323–2334. 21088012 10.1377/hlthaff.2010.0862

[11] Guessous I, Gaspoz JM, Theler JM, Wolff H (2012) High prevalence of forgoing healthcare for economic reasons in Switzerland: a population-based study in a region with universal health insurance coverage. Prev Med 55: 521–527. 22940614 10.1016/j.ypmed.2012.08.005

[12] Heisler M, Langa KM, Eby EL, Fendrick AM, Kabeto MU, et al (2004) The health effects of restricting prescription medication use because of cost. Med Care 42: 626–634. 15213486 10.1097/01.mlr.0000129352.36733.cc

[13] Chen J, Rizzo JA, Rodriguez HP (2011) The health effects of cost-related treatment delays. Am J Med Qual 26: 261–271. 21478458 10.1177/1062860610390352

[14] Heisler M, Wagner TH, Piette JD (2004) Clinician identification of chronically ill patients who have problems paying for prescription medications. Am J Med 116: 753–758. 15144912 10.1016/j.amjmed.2004.01.013

[15] Bihan H, Laurent S, Sass C, Nguyen G, Huot C, et al (2005) Association among individual deprivation, glycemic control, and diabetes complications: the EPICES score. Diabetes Care 28: 2680–2685. 16249539 10.2337/diacare.28.11.2680

[16] Vaucher P, Bischoff T, Diserens E-A, Herzig L, Meystre-Agustoni G, et al (2012) Detecting and measuring deprivation in primary care: development, reliability and validity of a self-reported questionnaire: the DiPCare-Q. BMJ open 2: e000692. 10.1136/bmjopen-2011-000692 PMC3274718 22307103

[17] Chandola T, Clarke P, Wiggins RD, Bartley M (2005) Who you live with and where you live: setting the context for health using multiple membership multilevel models. J Epidemiol Community Health 59: 170–175. 15650151 10.1136/jech.2003.019539 PMC1733009

[18] Paniz VM, Fassa AG, Maia MF, Domingues MR, Bertoldi AD (2010) Measuring access to medicines: a review of quantitative methods used in household surveys. BMC Health Serv Res 10: 146. 20509960 10.1186/1472-6963-10-146 PMC2890644

[19] Wisk LE, Witt WP (2012) Predictors of delayed or forgone needed health care for families with children. Pediatrics 130: 1027–1037. 23129081 10.1542/peds.2012-0668 PMC3507252

[20] Singh-Manoux A, Adler NE, Marmot MG (2003) Subjective social status: its determinants and its association with measures of ill-health in the Whitehall II study. Soc Sci Med 56: 1321–1333. 12600368 10.1016/s0277-9536(02)00131-4

[21] Rabin R, de Charro F (2001) EQ-5D: a measure of health status from the EuroQol Group. Ann Med 33: 337–343. 11491192 10.3109/07853890109002087

[22] Kemp A, Roughead E, Preen D, Glover J, Semmens J (2010) Determinants of self-reported medicine underuse due to cost: a comparison of seven countries. J Health Serv Res Policy 15: 106–114. 20203082 10.1258/jhsrp.2009.009059

[23] Anyaegbu G (2010) Using the OECD equivalence scale in taxes and benefits analysis. Economic & Labour Market Review 4: 49–54.

[24] Alexander GC, Casalino LP, Meltzer DO (2003) Patient-physician communication about out-of-pocket costs. JAMA 290: 953–958. 12928475 10.1001/jama.290.7.953

[25] Nederhof AJ (1985) Methods of coping with social desirability bias: A review. European Journal of Social Psychology 15: 263–280.

[26] Hausman A (2001) Taking your medicine: relational steps to improving patient compliance. Health Mark Q 19: 49–71. 11873456 10.1300/J026v19n02_05

[27] Alexander GC, Casalino LP, Tseng CW, McFadden D, Meltzer DO (2004) Barriers to patient-physician communication about out-of-pocket costs. J Gen Intern Med 19: 856–860. 15242471 10.1111/j.1525-1497.2004.30249.x PMC1492500

[28] Srivastava R (2011) Complicated lives—taking the social history. N Engl J Med 365: 587–589. 21848459 10.1056/NEJMp1106985

[29] Piette JD, Heisler M, Krein S, Kerr EA (2005) The role of patient-physician trust in moderating medication nonadherence due to cost pressures. Arch Intern Med 165: 1749–1755. 16087823 10.1001/archinte.165.15.1749

[30] Hardee JT, Platt FW, Kasper IK (2005) Discussing health care costs with patients: an opportunity for empathic communication. J Gen Intern Med 20: 666–669. 16050867 10.1111/j.1525-1497.2005.0125.x PMC1490152

[31] Brcic V, Eberdt C, Kaczorowski J (2011) Development of a tool to identify poverty in a family practice setting: a pilot study. Int J Family Med 2011: 812182. 22312547 10.1155/2011/812182 PMC3268233

[32] Gruen RL, Campbell EG, Blumenthal D (2006) Public roles of US physicians: community participation, political involvement, and collective advocacy. JAMA 296: 2467–2475. 17119143 10.1001/jama.296.20.2467

